# Nurses’ Willingness and Demand for Internet+Home Care Services and the Associated Factors in Municipal Hospitals in China: Cross-Sectional Survey

**DOI:** 10.2196/45602

**Published:** 2023-08-04

**Authors:** Jinghui Zhang, Sha Peng, Jianmei Hou, Guiyuan Ma, Yanhui Liu, Yuhua Fan, Lingxia Luo, Zhengkun Shi

**Affiliations:** 1 Teaching and Research Section of Clinical Nursing Xiangya Hospital Central South University Changsha China; 2 National Clinical Research Center for Geriatric Disorders Xiangya Hospital Central South University Changsha China; 3 Xiangya Nursing School Central South University Changsha China

**Keywords:** Internet+home care services, willingness, demand, clinical nurses, municipal hospitals

## Abstract

**Background:**

Developing Internet+home care (IHC) services is a promising way to address the problems related to population aging, which is an important global issue. However, IHC services are in their infancy in China. Limited studies have investigated the willingness and demand of nurses in municipal hospitals to provide IHC services.

**Objective:**

This study aims to investigate the willingness and demand of nurses in municipal hospitals in China to provide IHC services and analyze the factors to promote IHC development in China.

**Methods:**

This cross-sectional study used multistage sampling to recruit 9405 nurses from 10 hospitals in 5 regions of China. A self-designed questionnaire with good reliability and validity was used to measure nurses’ willingness and demand for providing IHC services. Data analysis used the chi-square test, Welch t test, binary logistic regression analysis, and multiple linear regression analysis.

**Results:**

Nurses were highly willing to provide IHC services and preferred service distances of <5 km and times from 8 AM to 6 PM. An individual share >60% was the expected service pay sharing. Job title, educational level, monthly income, and marital status were associated with nurses’ willingness to provide IHC services in binary logistic regression analysis. Supervising nurses were 1.177 times more likely to express a willingness to provide IHC services than senior nurses. Nurses with a bachelor's degree had a 1.167 times higher likelihood of expressing willingness to provide IHC services than those with a junior college education or lower. Married nurses were 1.075 times more likely to express a willingness than unmarried nurses. A monthly income >¥10,000 increased the likelihood of nurses’ willingness to provide IHC services, by 1.187 times, compared with an income <¥5000. Nurses’ total mean demand score for IHC services was 17.38 (SD 3.67), with the highest demand being privacy protection. Multiple linear regression analysis showed that job title, monthly income, and educational level were associated with nurses’ demand for IHC services. Supervising nurses (B=1.058, *P*<.001) and co-chief nurses or those with higher positions (B=2.574, *P*<.001) reported higher demand scores than senior nurses. Monthly incomes of ¥5000 to ¥10,000 (B=0.894, *P*<.001) and >¥10,000 (B=1.335, *P*<.001), as well as a bachelor's degree (B=0.484, *P*=.002) and at least a master's degree (B=1.224, *P*=.02), were associated with higher demand scores compared with a monthly income <¥5000 and junior college education or lower, respectively.

**Conclusions:**

Nurses in municipal hospitals showed a high willingness and demand to provide IHC services, with differences in willingness and demand by demographic characteristics. Accordingly, government and hospitals should regulate the service period, service distance, and other characteristics according to nurses’ willingness and demand and establish relevant laws and regulations to ensure the steady and orderly development of IHC services.

## Introduction

Population aging is an important global issue [1], particularly in China, where it has become a severe trend [2]. In 2020, China’s population aged ≥65 years reached 190.64 million people, accounting for 13.4% of the total population [3]. The current hospital-centered health care model struggles to meet the long-term needs of older adult patients [4]. Home care has become an effective way to address the aging population in countries like the United States, Canada, and Japan [5-10]. In China, home care services are primarily provided by community nurses. However, the existing level of community care and human resources is insufficient to meet the home care needs of the entire population [11]. To tackle this, the National Health Commission of China initiated pilot Internet+home care (IHC) services in 2019 [12]. IHC services provide an online application and offline service mode, connecting registered nurses from medical institutions with patients through an app. Patients place orders through an app; managers then use a web platform to dispatch orders based on factors like nurses’ qualifications, professionalism, and distance; and online nurses accept them during their off-duty time [13]. IHC services are a valuable supplement to traditional and transitional care [14], optimizing nursing talent, balancing nursing resource distribution, and meeting diverse patient needs. Additionally, this model offers flexibility for nurses, allowing them to arrange their working time and content, increase their income, and enhance their professional abilities [14].

Although IHC services in China have shown promise in some regions, they are still in their early stages. Challenges remain, including low nurse awareness, inadequate security measures, and imperfect service evaluation systems [15-18]. Addressing these issues is crucial for the further development and widespread adoption of IHC services in China. Municipal hospitals play a significant role in the health care landscape, accounting for a substantial proportion of all levels of hospitals in China [17]. A previous study showed that nurses in Chinese hospitals are strongly willing to provide IHC services [19], but no research has specifically focused on the willingness and demand of nurses in municipal hospitals. Given the pivotal role of nurses in municipal hospitals as the primary providers of IHC services, understanding their willingness and demand regarding IHC services is of considerable significance for the extensive development of IHC services in China.

Previous studies have employed different theoretical frameworks to examine the adoption and usage behaviors of electronic health care. For example, Kim et al [20] used the Technology Acceptance Model and Unified Theory of Acceptance and Use of Technology model to investigate the influencing factors of medical staff's usage behavior of mobile electronic medical records. Cho [21] employed the error correction model to study the postadoption behavior of health apps. Bastoni et al [22] used the non-adoption, abandonment, scale-up, spread, and sustainability (NASSS) framework to synthesize digital health implementation findings in informal care and home care across various populations.

Among the available frameworks, we selected the NASSS framework as a guide for our research due to its comprehensive synthesis of considerations for implementing, scaling, and sustaining digital health interventions [23]. This evidence-based and theory-informed framework encompasses both adoption and acceptance from stakeholders’ perspectives while also considering the wider context of implementation, including policy, regulations, and sociocultural factors [24].

In our study, we applied the NASSS framework to understand the willingness and demand of clinical nurses in municipal hospitals regarding IHC services. The 7 domains of the NASSS framework refer to the context of the specific health condition in which the technology is applied, characteristics of the technology, the added value of the technology, factors related to adopters, characteristics of the implementing organization, and wider institutional and sociocultural context of policy and regulations. The last domain refers to the relationship between the first 6 domains and the adaptation of the technology over time [23]. The framework's 7 domains provided a structured approach for our analysis. Specifically, we considered the context domain as the background of population aging, the technology domain encompasses the IHC services model, the value proposition domain focused on the potential benefits of IHC services for patients and nurses, the adopter domain involved the nurses and patients, and the organization domain considered the hospital management department and the IHC services platform. According to the NASSS framework, clinical nurses are the main provider of this new service model, so it is very important to fully understand their willingness and demand for providing IHC services. To the best of our knowledge, most previous studies that focused on investigating nurses’ willingness and demand to provide IHC services were qualitative studies [16,25], mainly concentrated in several provinces involved in pilot studies in China [18], and they had relatively small sample sizes. There is a lack of investigation into nurses’ willingness and demand in municipal hospitals to provide IHC services in China.

Guided by the NASSS framework, this study investigated the willingness and demand of clinical nurses in municipal hospitals in China regarding IHC services and analyzed the associated factors. Our findings may add value to the NASSS framework by providing empirical evidence and insights that inform the adopter, organization, and wider system domains, ultimately promoting the development of IHC services in China.

## Methods

### Study Design and Setting

We conducted a cross-sectional study from January 1, 2022, to August 31, 2022. The study setting was 10 hospitals in China. These hospitals were selected using a 3-stage random (using a random number table) sampling method. In stage one, 1 province-level division (ie, province, autonomous region, or direct-controlled municipality) was randomly selected from each of the 5 regions in Central, East, West, North, and South China. In this stage, 5 province-level divisions were selected. In stage two, 1 city or 1 administrative region was randomly selected from each of the 5 selected provinces, autonomous regions, or direct-controlled municipalities. In this stage, 5 cities or administrative regions were selected. In stage three, 2 hospitals were randomly selected from each of the 5 selected cities or administrative regions, totaling 10 hospitals.

### Participants

A total of 9405 nurses from 10 hospitals were randomly selected as the survey participants. This study recruited registered nurses (RNs) who met the following inclusion criteria: (1) aged ≥18 years but <60 years, (2) engaged in clinical work, (3) provided informed consent and participated voluntarily, and (4) had more than 5 years of clinical working experience or held a job title of “senior nurse.” RNs on medication for acute mental illness and those absent from work due to sick leave, personal leave, study abroad, or other reasons were excluded.

### Variables and Measures

#### Outcome Variables

The outcome variables for the present study were nurses’ willingness to provide IHC services and their demand for IHC services. Willingness refers to nurses’ inclination and preferences to engage in delivering IHC services, while demand represents their perceived need and desire for the implementation of such services. The independent variables were nurses’ gender, age, marital status, job title, educational level, and monthly income.

#### General Information Collection Form

A researcher-developed general information collection form was used to collect data related to independent variables, including gender, age, marital status, job title, educational level, and monthly income.

#### IHC Services Willingness and Demand Survey Questionnaire

The IHC Services Willingness and Demand Survey Questionnaire was used to measure nurses’ willingness to provide IHC services and their demand for such services. The questionnaire comprised 2 sections. Section one measured nurses’ willingness to provide IHC services using 4 items focusing on the willingness of nurses to provide IHC services and nurses’ preferences for service distance, service period, and service pay sharing. These items were answered with single-choice or dichotomous options (willing or unwilling). Section two measured nurses' demand for IHC services using 4 items, including their demand for formulating relevant laws, regulations, and an emergency plan, as well as their demand for implementing the IHC staff prejob training and assessment, demand for protecting the privacy of patients and nursing staff, and demand for establishing a unified standard charge and incorporating it into medical insurance. A 5-level Likert scale was used, ranging from 1 to 5 (from “not needed” to “very needed”). The total demand score was calculated by summing the scores of all items and dividing by the number of items, with a higher score indicating a higher level of demand.

#### Validity and Reliability of the IHC Services Willingness and Demand Survey Questionnaire

The questionnaire was developed through a literature search, a focus group discussion, Delphi expert consultation, and a preliminary survey.

A literature search was conducted to identify related literature to use to develop the pool of questionnaire items. We searched 4 databases, namely MEDLINE (via PubMed), Web of Science, Wanfang database, and China National Knowledge Infrastructure (CNKI), from their inception to December 30, 2021. The search terms were “Internet home care,” “home care,” “home based care,” “nurse,” and “factors.” A total of 233 studies were retrieved, comprising 224 from MEDLINE (via PubMed) and Web of Science, as well as 9 from the Wanfang database and CNKI. After removing duplicate literature and conducting screening based on titles and abstracts, 200 articles were excluded. The full text of the remaining 33 articles was read, and ultimately, 6 highly relevant articles [13,19,26-29] were included to develop the pool of questionnaire items. Additionally, 6 experts (ie, 2 nurses with experience in home care, 2 nurse researchers specializing in home care, and 2 nursing managers in home care settings) were invited for a focus group discussion. They provided feedback on the pool of items developed through the literature search and discussed modifications and additional suggestions for the item pool.

After the literature search and focus group discussion, a Delphi method was used to revise and validate the questionnaire. A panel of 20 experts from various fields, including security management, medical technology, nursing management, and the IHC platform, participated. Experts assessed the importance and relevance of each item using Likert scales and provided revision suggestions. Items with specific criteria, such as mean importance score ≤3.5, full importance score (ie, 5 points) ratio ≤0.8, coefficient of variation of importance score ≥0.25, or item-level content validity index <0.78 were removed from the item pool. After 2 rounds of the Delphi process, 8 items were finally retained for the IHC Services Willingness and Demand Survey Questionnaire. The scale content validity index value for the questionnaire was 0.799. A preliminary survey was conducted among 40 nurses to evaluate the reliability of the questionnaire. The Cronbach α value for this questionnaire was 0.748.

### Data Collection and Quality Control

Before the survey, the principal researchers communicated with the nursing department directors of the 10 recruited hospitals by telephone calls or on-site visits to obtain their approval and cooperation. The directors were provided with unified training, which included an explanation of study purpose, significance, methods, and inclusion and exclusion criteria for the participants. The directors then assisted with the recruitment of all potential eligible participants within their hospitals, ensuring adherence to principles of informed consent and voluntary participation.

After recruitment, a video conference was arranged by nursing department directors for the potential eligible participant. During the conference, the study purpose, significance, methods, and possible benefits and how to fill in the questionnaire were thoroughly explained. The data were collected using an electronic questionnaire administered via Wenjuanxing, ensuring the participants' privacy and data security. To maintain data integrity, participants were instructed to answer all questions as per the provided prompts and were prohibited from submitting multiple responses.

Following the questionnaire collection, 2 researchers double-checked the data, eliminating any invalid or incomplete questionnaires. To validate the data, a presurvey involving 30 nurses was conducted, with an average questionnaire completion time of approximately 5 minutes. Questionnaires with completion times of <5 minutes or with consistently uniform responses to all items were excluded. In total, 9506 questionnaires were collected, and 101 were eliminated, with an effective response rate of 98.9%.

### Ethical Considerations

The study was approved by the Medical Ethics Committee of Xiangya Hospital, Central South University, Changsha, China (Ethics Review and Approval Number 202012191). Informed consent was obtained from all participants before data collection, and the confidentiality of participants' clinical data and basic information was strictly maintained. The study adhered to the principles of the Declaration of Helsinki, ensuring ethical conduct throughout the research process.

### Statistical Analysis

Statistical analysis was performed using SPSS 25.0 (IBM Corp). Categorical data were described using frequency (count) and relative frequency (%), and continuous data were expressed as mean (SD). As the data did not follow a normal distribution and exhibited heteroscedasticity, we used the chi-square test (χ^2^) and Welch *t* test to analyze the differences in nurses’ willingness and demands for IHC services. To analyze associated factors, binary logistic regression (for nurses’ willingness) and multiple linear regression (for nurses’ demand) analyses were conducted using statistically independent variables identified from the univariate analyses. The entry and exclusion levels for variables in the regression equation were set at α=.05 and α=.10, respectively, and a backward likelihood ratio logistic regression method was applied. Statistically significant results were defined as *P*<.05 (2-sided).

## Results

### Study Participants

A total of 9405 nurses completed the survey, predominantly comprising women (9118/9405, 96.9%), married individuals (6640/9405, 70.6%), and those aged 30 years to 40 years (4200/9405, 44.7%). The distribution of job titles among participants included 5348 senior nurses, 3430 nurses in charge, and 627 individuals with the title of co-chief nurse or higher. The majority (6833/9405, 72.7%) had an undergraduate degree. More than one-half (5524/9405, 58.7%) of the participants reported a monthly income ranging from ¥5000 to ¥10,000, which is the average wealth of nurses in China's urban medical collective units (the average monthly income of China's nurses in urban medical service institutions is ¥8359 per month [30]; Table 1). A currency exchange rate of ¥1=US $0.14 is applicable.

**Table 1:** Demographic characteristics of the participants (N=9405).

Characteristics			Results, n (%)
**Gender**			
	Male	287 (3.1)	
	Female	9118 (96.9)	
**Marital status**			
	Unmarried	2574 (27.4)	
	Married	6640 (70.6)	
	Divorced or widowed	191 (2.0)	
**Age (years)**			
	<30	3910 (41.6)	
	30-40	4200 (44.7)	
	>40	1295 (13.7)	
**Job title**			
	Senior nurse	5348 (56.9)	
	Supervision nurse	3430 (36.5)	
	Co-chief nurse or above	627 (6.6)	
**Educational level**			
	Junior college or below	2536 (26.9)	
	Bachelor’s degree	6833 (72.7)	
	Master’s degree or higher	36 (0.4)	
**Monthly income (¥)**			
	<5000	2187 (23.3)	
	5000-10,000	5524 (58.7)	
	>10,000	1694 (18.0)	

### Nurses’ Willingness to Provide IHC Services and Associated Factors

More than 70% of the participants were willing to provide IHC services. The results showed that more than 70% (7263/9405, 77.2%) of the nurses expected the distance of IHC services to be <5 km from the departure place. In addition, 80% (7523/9405) of the nurses chose the time period from 8 AM to 6 PM for IHC services, and approximately 80% (7958/9405, 84.6%) of the nurses expected the service pay sharing to be an individual share >60% (Table 2).

Univariate analyses showed that nurses' overall willingness to provide IHC services was significantly different by their marital status, job title, educational level, and monthly income (all *P*<.05, Table 3). The binary logistic regression analysis showed that nurses holding a title of supervision nurse were 1.177 times more likely to express a willingness to provide IHC services than senior nurses. Nurses with a bachelor’s degree had a 1.167 times higher likelihood of being willing to provide IHC services than nurses with a junior college education or lower. Married nurses demonstrated a 1.075 times greater likelihood of expressing a willingness to provide IHC services than unmarried nurses. Additionally, nurses with a monthly income exceeding ¥10,000 were 1.187 times more likely to express a willingness to provide IHC services than nurses with a monthly income below ¥5000 (Table 4).

**Table 2:** Nurses’ willingness to provide Internet+home care services (N=9405).

Items			Results, n (%)
**Overall willingness**			
	Willing	6673 (71.0)	
	Unwilling	2372 (29.0)	
**Preference for service distance**			
	<5 km from the departure location	7263 (77.2)	
	>5 km from the departure location	2142 (22.8)	
**Preference for service period**			
	8 AM to 6 PM	7523 (80)	
	6 PM to 8 PM	1882 (20)	
**Preference for service pay sharing**			
	Individual share <60%	1447 (15.4)	
	Individual share >60%	7958 (84.6)	

**Table 3:** Nurses’ willingness to provide Internet+home care services by different demographic characteristics (N=9405).

Characteristics		Overall willingness		Chi-square (df)	*P* value
		Willing, n (%)	Unwilling, n (%)		
**Gender**				1.288 (9398)	.13
	Male	228 (79.4)	59 (20.6)		
	Female	6964 (76.4)	2154 (23.6)		
**Marital status**				45.508 (9398)	<.001
	Unmarried	1913 (74.3)	661 (25.7)		
	Married	5225 (78.7)	1415 (21.3)		
	Divorced or widowed	177 (92.7)	14 (7.3)		
**Age (years)**				28.707 (9398)	<.001
	<30	2898 (74.1)	1012 (25.9)		
	30-40	3324 (79.1)	876 (20.9)		
	>40	997 (77)	298 (23)		
**Job title**				12.405 (9398)	.002
	Senior nurse	4149 (77.6)	1199 (22.4)		
	Supervision nurse	2682 (78.2)	748 (21.8)		
	Co-chief nurse or higher	525 (83.7)	102 (16.3)		
**Educational level**				35.727 (9398)	<.001
	Junior college or lower	1880 (74.1)	656 (25.9)		
	Bachelor’s degree	5444 (79.7)	1389 (20.3)		
	Master’s degrees or higher	32 (88.9)	4 (11.1)		
**Monthly income (¥)**				89.702 (9398)	<.001
	<5000	411 (18.8)	1776 (81.2)		
	5000-10,000	4338 (78.5)	1186 (21.5)		
	>10,000	1505 (88.8)	189 (11.2)		

**Table 4:** Binary logistic regression of nurses’ willingness to provide Internet+home care services.

Variables		B	SE	Wald	*P* value	EXP(B)	95% CI for EXP(B)
(Constant)		1.846	0.471	15.35	<.001	—^a^	—
**Job title**							
	Senior nurse^b^	—	—	—	—	—	—
	Supervision nurse	0.163	0.067	5.861	.02	1.177	1.032-1.344
	Co-chief nurse or higher	0.292	0.126	5.263	.02	1.336	1.043-1.712
**Educational level**							
	Junior college or lower^b^	—	—	—	—	—	—
	Bachelor’s degree	0.154	0.055	8.03	.005	1.167	1.049-1.299
	Master’s degrees or higher	0.323	0.407	0.63	.04	1.381	1.306-1.976
**Marital status**							
	Unmarried^b^	—	—	—	—	—	—
	Married	0.073	0.065	1.244	.04	1.075	1.035-1.852
	Divorced or widowed	0.330	0.184	3.221	.07	1.391	0.97-1.996
**Monthly income (¥)**							
	<5000^b^	—	—	—	—	—	—
	5000-10,000	0.071	0.058	1.458	.23	1.074	0.957-1.204
	>10,000	0.169	0.079	4.545	.03	1.187	1.017-1.386

^a^Not applicable.

^b^Reference group.

### Nurses’ Demand for IHC Services and Associated Factors

The results showed the nurses’ total mean demand score for providing IHC services was 17.38 (SD 3.67), and the 3 most demanded items were “demand for protecting the privacy of patients and nursing staff” (mean 4.44, SD 0.968), “demand for establishing a unified standard charge and incorporating it into medical insurance” (mean 4.35, SD 1.014), and “demand for formulating relevant laws, regulations, and emergency plans” (mean 4.32, SD 1.021; Table 5).

Univariate analyses showed that nurses' total demand score was significantly different by their gender, marital status, job title, educational level, and monthly income (all *P*<.05, Table 6). Multiple linear regression showed that job title, monthly income, and educational level could influence municipal hospital nurses’ demand for providing IHC services (see Multimedia Appendix 1 for the dummy variable assignment), which explained 2.5% of the total variation. Nurses holding a job title of supervision nurse (B=1.058, *P*<.001) or co-chief nurse or higher (B=2.574, *P*<.001) reported a higher total demand score, indicating a higher level of demand for IHC services, than those with a job title of senior nurse. Nurses with monthly incomes of ¥5000 to ¥10,000 (B=0.894, *P*<.001) or >¥10,000 (B=1.335, *P*<.001) reported higher total demand scores, indicating a higher level of demand for IHC services, than those with monthly incomes <¥5000. Nurses holding a bachelor’s degree (B=0.484, *P*=.002) or at least a master’s degree (B=1.224, *P*=.02) reported higher total demand scores, indicating a higher level of demand for IHC services, than those with an educational level of junior college or lower (Table 7).

**Table 5:** Demand from nurses to provide Internet+home care (IHC) services.

Category		Total demand score, mean (SD)	Demand for formulating relevant laws, regulations, and an emergency plan, mean (SD)	Demand for implementing the IHC staff prejob training and assessment, mean (SD)	Demand for protecting the privacy of patients and nursing staff, mean (SD)	Demand for establishing a unified standard charge and incorporating it into medical insurance, mean (SD)	

Entire sample		17.38 (3.67)	4.32 (1.02)	4.27 (1.02)	4.44 (0.97)	4.35 (1.01)	
**Gender**							
	Male	16.74 (4.27)	4.19 (1.14)	4.15 (1.13)	4.25 (1.09)	4.15 (1.17)	
	Female	17.40 (3.65)	4.33 (1.02)	4.28 (1.02)	4.45 (0.96)	4.35 (1.09)	
**Marital status**							
	Unmarried	16.99 (3.89)	4.25 (1.07)	4.17 (1.08)	4.36 (1.03)	4.21 (1.08)	
	Married	17.53 (3.56)	4.35 (1.00)	4.31 (0.99)	4.47 (0.94)	4.40 (0.98)	
	Divorced or widowed	17.69 (4.12)	4.41 (1.07)	4.37 (1.09)	4.49 (1.07)	4.42 (1.13)	
**Age (years)**							
	<30	17.02 (3.83)	4.23 (1.06)	4.19 (1.06)	4.37 (1.01)	4.23 (1.07)	
	30-40	17.57 (3.58)	4.37 (0.99)	4.32 (1.00)	4.48 (0.94)	4.41 (0.98)	
	>40	17.87 (3.36)	4.45 (0.95)	4.38 (0.97)	4.54 (0.89)	4.50 (0.91)	
**Job title**							
	Senior nurse	17.00 (3.86)	4.23 (1.07)	4.18 (1.07)	4.36 (1.02)	4.24 (1.07)	
	Supervision nurse	17.79 (3.41)	4.42 (0.96)	4.37 (0.96)	4.52 (0.90)	4.47 (0.92)	
	Co-chief nurse or higher	18.45 (2.82)	4.62 (0.81)	4.53 (0.86)	4.67 (0.76)	4.63 (0.80)	
**Educational level**							
	Junior college or lower	16.93 (3.91)	4.20 (1.07)	4.18 (1.06)	4.34 (1.04)	4.21 (1.09)	
	Bachelor’s degree	17.55 (3.56)	4.37 (0.99)	4.30 (1.01)	4.48 (0.94)	4.40 (0.98)	
	Master’s degree or higher	18.31 (3.38)	4.50 (0.94)	4.53 (0.91)	4.69 (0.82)	4.58 (0.87)	
**Monthly income (¥)**							
	<5000	16.66 (4.03)	4.13 (1.11)	4.11 (1.09)	4.28 (1.07)	4.15 (1.11)	
	5000-10,000	17.50 (3.58)	4.35 (0.99)	4.30 (1.01)	4.47 (0.95)	4.38 (0.99)	
	>10,000	17.93 (3.32)	4.47 (0.95)	4.41 (0.95)	4.56 (0.87)	4.49 (0.94)	

**Table 6:** Nurses’ total demand score to provide Internet+home care services by different demographic characteristics.

Category			Total demand score, mean (SD)		Welch		*P* value
**Gender**					16.81		.01
	Male	16.74 (4.27)					
	Female	17.40 (3.65)					
**Marital status**					18.701		<.001
	Unmarried	16.99 (3.89)					
	Married	17.53 (3.56)					
	Divorced or widowed	17.69 (4.12)					
**Age (years)**					36.816		<.001
	<30	17.02 (3.83)					
	30-40	17.57 (3.58)					
	>40	17.87 (3.36)					
**Job title**					27.194		<.001
	Senior nurse	17.00 (3.86)					
	Supervision nurse	17.79 (3.41)					
	Co-chief nurse or higher	18.45 (2.82)					
**Educational level**					24.98		<.001
	Junior college or lower	16.93 (3.91)					
	Bachelor’s degree	17.55 (3.56)					
	Master’s degrees or higher	18.31 (3.38)					
**Monthly income (¥)**					34.628		<.001
	<5000	16.66 (4.03)					
	5000-10,000	17.50 (3.58)					
	>10,000	17.93 (3.32)					

**Table 7:** Multiple linear regression analysis of nurses’ demand to provide Internet+home care services (F9401=25.281, R=0.162, R2=0.026, adjusted R2=0.025).

Variables		B	SE	β	*t* (df)	*P* value
(Constant)		27.738	0.164	—^a^	169.114 (1)	<.001
**Job title**						
	Senior nurse^b^	—	—	—	—	—
	Supervision nurse	1.058	0.185	0.082	5.703 (9401)	<.001
	Co-chief nurse or higher	2.574	0.340	0.103	7.565 (9401)	<.001
**Monthly income (** **¥)**						
	<5000^b^	—	—	—	—	—
	5000-10,000	0.894	0.166	0.071	5.386 (9401)	<.001
	>10,000	1.335	0.219	0.083	6.108 (9401)	<.001
**Educational level**						
	Junior college or lower^b^	—	—	—	—	—
	Bachelor’s degree	0.484	0.155	0.035	3.124 (9401)	.002
	Master’s degrees or higher	1.224	1.038	0.012	1.179 (9401)	.02

^a^Not applicable.

^b^Reference group.

## Discussion

### Principal Findings

This study found that most nurses express a willingness to provide IHC services, which is consistent with the review conducted by Sheng et al [13]. The minority showed little interest, which may be explained by the fact that IHC services have not yet been implemented nationwide; only some pilot work has been conducted in some cities [12]. A significant proportion of nurses preferred to provide IHC services from 8 AM to 6 PM, within a service range not exceeding 5 km from their departure location. These findings align with previous research conducted by Li et al [19]. Nurses preferred daytime hours (8 AM to 6 PM) for IHC services within a shorter service range, potentially due to safety considerations [31]. Daytime hours offer higher safety levels, with ample staff and resources available to ensure safety, and a smaller service range contributes to enhanced safety during travel. According to Khamisa et al [32]*,* workplace safety risks have an impact on nurses’ job satisfaction. By enhancing safety measures during the IHC service process, nurses’ satisfaction with IHC services can be enhanced [32], thereby potentially improving their willingness to provide such services. Furthermore, a majority of nurses expressed a preference for a service pay-sharing model with an individual share exceeding 60%. This finding aligns with a previous study highlighting the significance of economic income derived from IHC services as an important influencing factor for nurses engaging in IHC services and multipoint practice [33]. Based on these findings, it is recommended that the government establish an Internet Nursing Alliance to streamline service dispatch based on patients’ needs and home addresses, thereby ensuring control over service distance and time. Furthermore, implementing uniform and reasonable pricing for IHC services can serve as a stimulus for nurses' enthusiasm toward providing these services. In addition, the platform is recommended to provide nurses with the security of knowing their safety and interests are addressed and to purchase personal accident insurance and medical liability insurance for nurses [34].

Our study revealed that married nurses exhibited a greater willingness to provide IHC services than unmarried nurses, aligning with the findings reported by Ma et al [17]. It is possible that the higher willingness among married nurses stems from their increased economic responsibilities, such as housing loans and caregiving for children or older family members. Although married nurses may have less free time and flexibility in their schedules [35], they may be more inclined to offer IHC services during nonstandard working hours to alleviate financial pressures and secure additional income. Furthermore, our study found that nurses with higher education levels and higher job titles demonstrated a greater willingness to provide IHC services than those with a junior college education or lower or those holding a job title of senior nurse, which was consistent with the results reported by Ma et al [17]. The plausible explanations were that nurses with higher job titles and educational levels usually have an extensive knowledge base, extensive clinical experience, and heightened professional confidence; therefore, they may exhibit a greater willingness to utilize their expertise to assist patients by providing IHC services. Interestingly, our study also revealed that nurses with higher monthly incomes were more willing to provide IHC services than those with a monthly income less than ¥5000. This finding contrasts with the expectation that nurses with lower incomes may express a greater willingness to engage in IHC services, as suggested by previous research emphasizing the economic benefits derived from such services [33]. The reason for this phenomenon could potentially be that nurses with a higher monthly income are more inclined to choose high-income jobs because providing IHC services is considered a job that can yield a higher income in a short period of time. However, the underlying reasons for this finding remain unclear. Further research, particularly qualitative investigations, is needed to explore the motivations and factors influencing the willingness of nurses with higher incomes to provide IHC services.

Consistent with the high level of willingness, our study found that the demand by nurses for IHC services is substantial, which was specifically demonstrated in the following areas: “demand for formulating relevant laws, regulations, and emergency plans,” “demand for implementing the IHC staff prejob training and assessment,” “demand for protecting the privacy of patients and nursing staff,” and “demand for establishing a unified standard charge and incorporating it into medical insurance.” Although previous studies have acknowledged the high demand for training in IHC services among nurses [18,29,36], our study is the first to identify their high demands in these 4 specific areas. By addressing these specific needs, such as comprehensive legal frameworks, effective training programs, privacy protection measures, and standardized charge systems to optimize the delivery of IHC services, we can help create an enabling environment that promotes the growth and success of IHC services. This, in turn, benefits both nurses and the patients they serve. Overall, our study contributes to the understanding of nurses' multifaceted demands in IHC services, highlighting the importance of addressing these specific areas to optimize the delivery of care and ensure the effectiveness of IHC programs.

Finally, our study revealed that nurses with higher job titles, higher educational levels, and higher monthly incomes expressed a significant demand for IHC care compared with their counterparts with lower job titles, lower educational levels, and lower monthly incomes. Because most of the people with higher titles and more education have longer working hours, extensive work experience, and are able to calmly and stably handle various unexpected events that may arise [17], they have given sufficient consideration to their demands, such as the requirements for improving the qualifications of nurses providing IHC services and developing complete regulations and laws [37]. Consistently, previous studies reported that professional title, educational level, and mortgage or car loan were associated with nurses’ demand for IHC services [13,17]. It is recommended that relevant departments prioritize the development of nurses with higher education and higher professional titles to provide IHC services, then observe the results and take some action to promote the development of IHC services. It is an interesting finding that nurses with a higher monthly income have a higher demand for providing IHC services, which has not been reported before. To avoid the loss of high-quality resources, hospitals usually increase the salary of nurses with higher education and professional titles. Such nurses have higher monthly incomes and higher demand for IHC services. IHC services require comprehensive quality of nursing personnel. Because of the environment and change in practice, it is challenging to meet the needs of IHC services based on prior knowledge and skills; therefore, vocational education and training should be conducted to prepare for the IHC services program, which can also promote nurse development and enhance their sense of career benefits.

### Future Recommendations

The NASSS framework provides a comprehensive framework to support systematic assessments of health care innovation, incorporating various perspectives on health and illness, technology adoption, organizational change, and health service design [38]. According to the NASSS framework and based on this study’s findings, we put forward the following considerations. First, from the perspective of technology adopters, nursing managers can start with nurses with higher job titles and higher educational levels and gradually carry out IHC services, which is also in line with the provisions in the Chinese IHC services trial document stating that IHC nurses must have more than 5 years of nursing experience [3]. At the same time, the management department can also conduct knowledge lectures, knowledge competitions, and training courses on IHC services, which can improve nurses' understanding of the benefits of IHC and willingness to provide IHC services. Second, we can use some technologies in the IHC services. The study by Liljeroos and Arkkukangas [37] indicated that suitable adoption of devices and technologies is important in facilitating care delivery. Adequate patient and caregiver training and easy-to-use devices, such as wearables for remote vital sign monitoring, have been shown to result in greater satisfaction, improved ability to use the device, and better overall functioning [37], which is related to the technology areas mentioned in the NASSS framework [39]. Third, based on our research, we recommend that medical institutions should establish clear IHC service management norms, an evaluation system for service work quality, service process and content, an evaluation system for the service object, and emergency plans. The management center of the platform can formulate the personal electronic medical records of patients at home, which is convenient for the whole nursing management process, such as tracking, follow-up, and conducting a satisfaction survey [40]. This approach advocates exchanging patients' personal information and medical information with the platform of medical institutions, which is related to the organization areas mentioned in the NASSS framework. Fourth, managers should standardize the performance evaluation system of IHC services, reasonably assign orders, and improve nurses’ individual service pay to share. We also recommend that management departments formulate unified standard charges or use the market bargaining mechanism to establish a price and related payment guarantee mechanism to protect the rights and interests of nursing staff and patients and establish a medical insurance system with basic medical insurance as the main body, other forms of medical insurance, and commercial insurance as supplements to promote the IHC services in a planned and step-by-step manner. Finally, we can use the IHC alliance form, according to the location of patients and hospitals and according to the principle of nearby selection, thus reasonably arranging and allocating orders to nurses with internet nursing qualifications in allied hospitals. This can not only save time and cost but also optimize the allocation of medical and nursing resources, promote the implementation of graded diagnosis and treatment, and thus improve the penetration rate of IHC services in China.

### Strengths and Limitations

This study explored for the first time the willingness and demand of clinical nurses in municipal hospitals in China regarding IHC services and analyzed the associated factors. The questionnaire was developed through a literature search, a focus group discussion, Delphi expert consultation, and a preliminary survey. Moreover, the very large sample size ensured reliable results. However, the cross-sectional study and potential regional imbalance in the sample may limit the generalizability of the findings. Future studies may consider expanding the investigation area to include a more diverse range of hospitals and regions, increasing the sample size to improve the representativeness of the findings, and conducting qualitative studies to gain a deeper understanding of the factors influencing nurses' willingness and demand regarding IHC services.

### Conclusions

As an emerging service model, IHC services are an organic combination of home nursing service and Internet technology. The model draws from both medical and health behavior and information behavior. Therefore, this study used the NASSS framework to understand the influencing factors for IHC service implementation in the entire research field, which strengthens the explanatory power of theoretical models on the adoption of IHC services. This theoretical framework provides a relatively comprehensive theoretical reference for other studies related to IHC services. With the development of the new nursing model of IHC, high-quality nursing resources are no longer concentrated in tertiary hospitals but can flow to smaller hospitals. The results of this study show that the willingness of clinical nurses in municipal hospitals to provide IHC services is at a high level and there is a high demand for improving the rules and regulations of IHC services. Nurse managers should strengthen the training of nurses to provide IHC services and improve service pay-sharing for IHC services. At the same time, relevant departments can formulate laws and regulations, refine and implement them, clarify responsibilities and risks, reasonably allocate medical staff and medical resources, and promote the development of the multipoint practice of nurses. At the same time, the development experience and mode of IHC abroad, such as appropriately expanding the service authority of on-site nurses, improving the evaluation system for the quality of on-site nursing services, and improving the medical insurance payment system, should be used for further education. Furthermore, health care financing should treat home hospitalization equal to ward hospitalization, allowing equivalent subsidies and insurance coverage to promote the better development of IHC services.

## Appendix

Multimedia Appendix 1 Assignment of the independent variables in the multiple linear regression analysis.

## Abbreviations

CNKI: China National Knowledge Infrastructure
IHC: Internet+home care
NASSS: non-adoption, abandonment, scale-up, spread, and sustainability
RN: registered nurse

## Data Availability

The data sets generated or analyzed during the current study are not publicly available due to the terms of consent and assent to which the participants agreed but are available from the corresponding author upon reasonable request.
