# Supplementary material for: Nurses’ Willingness and Demand for Internet+Home Care Services and the Associated Factors in Municipal Hospitals in China: Cross-Sectional Survey
Source: J Med Internet Res. 2023 Aug 4;25:e45602. doi: 10.2196/45602 (PMC10439466; doi:10.2196/45602)
Supplement: Multimedia Appendix 1 [file jmir_v25i1e45602_app1.docx]

**Table S1.** Assignment of the independent variables in the multiple linear regression analysis.

| Variables | Variable code | Assignment |
| --- | --- | --- |
| Gender | X1 | 1=male, 2=female |
| Marital Status | X2 | Unmarried (X2a=0, X2b=0, X2c=0, as a reference group) married (X2a=0, X2b=1, X2c=0) divorced or widowed (X2a=0, X2b=0, X2c=1) |
| Age | X3 | <30 (X3a=0, X3b=0, X3c=0, as a reference group) 30-40 (X3a=0, X3b=1, X3c=0) >40 (X3a=0, X3b=0, X3c=1) |
| Job title | X4 | Senior Nurse (X4a=0, X4b=0, X4c=0, as a reference group)  Supervision nurse (X4a=0, X4b=1, X4c=0） Co-chief Nurse or above (X4a=0，X4b=0, X4c=1) |
| Educational level | X5 | Junior college or below (X5a=0, X5b=0, X5=0，as a reference group) Bachelor’s degree (X5a=0, X5b=1, X5c=0) Master’s degrees or above (X5a=0, X5b=0, X5c=1) |
| Monthly income | X6 | <5000 Chinese yuan (X5a=0, X5b=0, X5=0, as a reference group) 5000-10000 Chinese yuan (X5a=0, X5b=1, X5=0) >10000 Chinese yuan (X5a=0, X5b=0, X5=1) |
